# Systematic review and meta-analysis of Endostar (rh-endostatin) combined with chemotherapy versus chemotherapy alone for treating advanced non-small cell lung cancer

**DOI:** 10.1186/1477-7819-10-170

**Published:** 2012-08-24

**Authors:** Rong Biaoxue, Yang Shuanying, Li Wei, Zhang Wei, Ming Zongjuan

**Affiliations:** 1 Department of Respiratory Medicine, The Second Affiliated Hospital of Xi’an Jiaotong University, 157 Xi-Wu Road, Xi’an, Shaanxi, 710004, China; 2 Department of Thoracic Surgery, The Second Affiliated Hospital of Xi’an Jiaotong University, 157 Xi-Wu Road, Xi’an, Shaanxi, 710004, China

**Keywords:** Recombinant human endostatin, Endostar, YH-16, Lung cancer, First-line chemotherapy, Meta-analysis

## Abstract

**Background:**

Many studies have investigated the efficacy of Endostar combined with platinum-based doublet chemotherapy (PBDC) versus PBDC alone for treating advanced non-small cell lung cancer (NSCLC). This study is a meta-analysis of available evidence.

**Methods:**

Fifteen studies reporting Endostar combined with PBDC versus PBDC alone for treating advanced NSCLC were reviewed. Pooled odds ratios and hazard ratio with 95% confidence intervals were calculated using either the fixed effects model or random effects model.

**Results:**

The overall response rate (ORR) and disease control rate (DCR) of Endostar combined with PBDC for treating NSCLC were significantly higher than those of PBDC alone, with 14.7% and 13.5% improvement, respectively (*P* < 0.00001). In addition, the time to progression (TTP) and quality of life (QOL) were improved after the treatment of Endostar combined with PBDC (*P* < 0.00001). The main adverse effects found in this review were hematological reactions, hepatic toxicity, and nausea/vomiting. Endostar combined with PBDC had a similar incidence of adverse reactions compared with PBDC alone (*P* < 0.05).

**Conclusions:**

Endostar combined with PBDC was associated with higher RR, DCR, and TTP as well as superior QOL profiles compared with PBDC alone. Endostar combined with PBDC had a similar incidence of adverse reactions compared with PBDC alone.

## Background

Lung cancer is one of the most common malignancies in the world. More than one million new cases are reported globally every year, and the five-year survival rate is less than 15% [1]. Non-small cell lung cancer (NSCLC) comprises 80% to 85% of lung cancer cases. Generally, 25% to 30% of NSCLC patients are in locally advanced stage upon diagnosis, and 40% to 50% of patients have distant metastases, losing their opportunities for surgery [2]. The current first-line chemotherapy options for patients with advanced NSCLC, such as the combination of platinum-based agents with paclitaxel, gemcitabine, vinorelbine, or docetaxel, have substantial toxicity and seem to have reached a plateau in terms of efficacy [3,4]. The use of cytotoxic chemotherapy is associated with a response rate (RR) of 20% to 35% and a median survival time of 10 to 12 months among patients with advanced NSCLC [5]. Novel regimens are needed to improve outcome, and the development of more effective therapies remains challenging.

In recent years, the clinical application of antiangiogenic therapy has brought promise for the treatment of NSCLC and has become an important addition in the treatment of tumor invasion and metastasis. In 1997, Folkman *et al*. first identified endostatin, the 20 kD internal fragment of the carboxyterminus of collagen XVIII, in the conditioned media of hemangioendothelioma cells as an antiangiogenic molecule [6]. The direct target of endostatin is the new capillary endothelial cells around the tumor. Endostar (YH-16), a novel recombinant human endostatin expressed and purified in *Escherichia coli*, was approved by China’s State Food and Drug Administration (SFDA) for the treatment of NSCLC in 2005. Compared with rh-endostatin reported in previous literature, an additional nine-amino acid sequence (MGGSHHHHH) was added at the N-terminal of the protein, which resulted in the formation of a six-histidine tag that could be chelated with metal ions such as Ni^2+^ with a relatively high affinity. These changes simplified the purification and improved the stability of the protein [7,8]. However, the effect of the structural changes on the antiangiogenic efficacy, including the mechanism of action, remains unknown.

To date, several studies discuss the efficacy and safety of Endostar in treating advanced lung cancer. Authentic assessment of Endostar treatment in lung cancer is important and urgent. The current study presents a systematic review to quantify the toxicities and clinical benefits of Endostar combined with platinum-based doublet chemotherapy (PBDC) versus chemotherapy alone for treating advanced NSCLC.

## Methods

### Search strategy and data extraction

An electronic search of scientific literature published in the databases of MEDLINE/PubMed, EMBASE, Cochrane Library, Science Citation Index, Current Controlled Trials, and CNKI was performed using free text and Medical Subject Heading terms such as ‘non-small cell lung cancer’, ‘NSCLC’, ‘lung adenocarcinoma’, ‘lung cancer’, ‘lung squamous carcinoma’, ‘rh-endostatin’, ‘endostatin’, ‘chemotherapy’, ‘Endostar’, and ‘recombinant human endostatin injection’. The search period was from the start of each database up to July 2012 without language restrictions. Moreover, a manual revision of the bibliographical references of the selected articles was done. In addition to the database search, papers were also identified by personal contact with the authors using email and telephone as necessary. The extracted data are summarized as follows: (i) general information, including the title, author, publication date, and literature sources; (ii) design and implementation, including the type of design, research and follow-up time, interventions, measurement indicator, the number of lost and processed samples; and (iii) outcome indicators, including RR, disease control rate (DCR), one-year survival rate (OYS), time to progression (TTP), quality of life (QOL), and adverse effects (AEs).

### Criteria for inclusion and exclusion

Meta-analysis inclusion criteria were as follows: (i) trials must compare Endostar combined with PBDC to PBDC alone for treating advanced NSCLC; (ii) patients in the studies meeting the first inclusion criteria must be diagnosed and confirmed by cytology and pathology; (iii) age and gender must not be restricted; (iv) must report on at least one of the outcome measures mentioned in the succeeding portion of this study; (v) randomized phase II and III studies were eligible if fully published; and (vi) the total number of cases must be greater than or equal to 40.

Abstracts, letters, editorials and expert opinions, reviews without original data, and case reports were excluded. The following studies were also excluded: (i) those with no clearly reported outcomes of interest; (ii) those evaluating patients with other types of malignant tumors and did not contain a distinct group of patients with NSCLC; and (iii) studies lacking control groups.

### Type of trial design, interventions, and indicators to determine efficacy

Trial design: randomized controlled trials of Endostar combined with PBDC versus PBDC for treating advanced NSCLC. Type of interventions: (i) Endostar + PBDC vs. PBDC; (ii) Endostar substituted one or more drugs of PBDC vs. PBDC; (iii) Endostar + PBDC A vs. PBDC B; and (iv) Endostar + PBDC + radiotherapy vs. PBDC + radiotherapy. Efficacy indicators: overall response rate (ORR), DCR, OYS, TTP, QOL, and AEs (according to the toxicity criteria of WHO).

### Methodological quality assessment

The methodological quality for randomized controlled trials (RCTs) was assessed using the criteria from the Cochrane Handbook for Systematic Reviews of Interventions (Version 5.0.1). The quality of trials was categorized into low risk of bias, unclear risk of bias, or high risk of bias. This categorization was according to the risk for each important outcome within included trials, including adequacy of the generation of allocation sequence, allocation concealment, blinding, and the presence of incomplete outcome data, selective outcome, or other sources of bias. The intention-to-treat (ITT) analysis was also assessed for the randomized controlled trials included in the present meta-analysis [9,10].

### Statistical analysis

To assess the efficacy and safety of Endostar combined with PBDC versus PBDC alone for treating advanced NSCLC, two different meta-analysis approaches were used: a fixed effects model and a random effects model. Dichotomous variables were analyzed using estimation of odds ratios (OR) and hazard ratio (HR) with a 95% confidence interval (95% CI). The overall effect was tested using Z-scores, with significance being set at *P* < 0.05. Pooled effect was calculated using either the fixed effects model or random effects model. Heterogeneity was evaluated through chi-square and I^2^. In the absence of statistically significant heterogeneity, the fixed effects method was used to combine the results. When heterogeneity was confirmed, the random effects method was used. Meta-regression was done to evaluate whether results were different between two groups. Sensitivity was analyzed by omitting each study from the estimated pool conducted at each step. Finally, publication bias was evaluated using funnel plots, the Egger’s test, and the Begg’s test. Statistical analyses were performed using SPSS (SPSS Institute, version 15.0, Chicago, USA), RevMan 4.2 (The Cochrane Collaboration), and Stata version 12.0 (Stata Corporation, TX, USA). All *P*-values were two-sided, and *P* < 0.05 was considered to indicate statistical significance.

## Results

### Selection of studies

**Figure 1:**
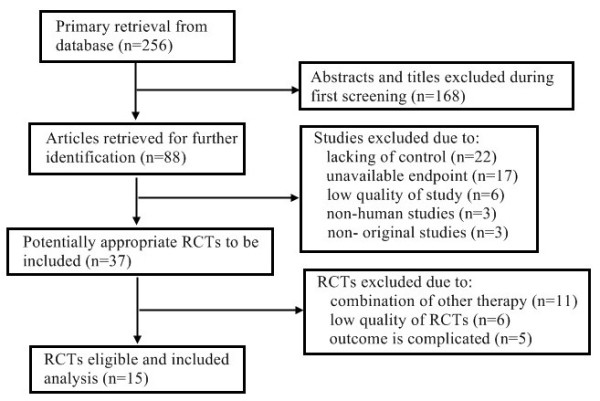
**Flow chart of literature search.** RCTs, randomized controlled trials.

Our systematic search identified 256 potentially relevant abstracts, of which 88 were identified as requiring full-text article retrieval. Close screening of these 88 studies excluded 68 because of the following reasons: limited cases (n < 40), non-human studies, and some received Endostar therapy without a parallel control. Finally, 15 studies published between 2005 and 2012 matched the inclusion criteria and were therefore included [11-25] (Figure 1). A database was established according to the extracted information from each selected paper. Table 1 shows the baseline demographic factors of the patients. The eligible studies included 1953 patients, of whom 621 were women and 1332 were men. The sample sizes oscillated between 46 [18] and 486 patients [11], and the age of the patients mainly concentrated at the range of 40 to 70 years old, with the youngest at 18 years old [11] and the oldest at 78 years old [20]. 

**Table 1:** Patient characteristics of the clinical trials reviewed

**Study**	**N**	**F/M**	**Age (Mean)**	**Histology (N)**	**TNM Stage (N)**	**Quality of Life**	**Invention Group (N)**	**Group 1 (N)**	**Group 2 (N)**	**End point**
Wang JW 2005 [11]	486	140/346	18-75	SCC (129) LAC (165) Others (28)	IIIA (51) IIIB (81) IV (185)	ECGO	NPE versus NP + placebo	322	164	RR, DCR, TTP, SI, AEs
Yang L 2005 [12]	87	28/59	37-76	SCC (34) LAC (50) Others (3)	III (32) IV (55)	ECGO	NPE versus NP	57	33	RR, DCR, SI, AEs
Cai L 2007 [13]	71	25/46	NA	SCC (27) LAC (32) Others (12)	IIIA (9) IIIB (33) IV (29)	KPS	NPE versus NP	39	32	RR, DCR, AEs
Mu HY 2009 [14]	62	22/40	42-75	SCC (28) LAC (34)	IIIB (28) IV (34)	KPS	NPE versus NP (16) TPE versus TP (6) PPE versus PP (8)	32	30	RR, DCR, SI, AEs
Liao HY 2009 [15]	85	32/53	36-72	SCC (36) LAC (49)	IIIB (32) IV (53)	KPS	GPE versus GP	30	55	RR, DCR, OYS, AEs
Zang T 2009 [16]	104	36/68	42-68	NA	IIIB (58) IV (46)	ECGO	GPE versus GP	48	56	RR, DCR, SI, AEs
Liu J 2009 [17]	62	17/45	29-68	SCC (38) LAC (24)	III (37) IV (25)	KPS	NPE + RT versus NP + RT	31	31	RR, DCR, TTP, AEs
Ma JB 2009 [18]	46	11/35	38-73	SCC (31) LAC (15)	IIIA (26) IIIB (20)	ECGO	NPE + RT versus NP + RT	23	23	RR, DCR, TTP, AEs
Shi GY 2009 [19]	462	162/300	20-74	SCC (190) LAC (252) Others (20)	IIIA (92) IIIB (144) IV (226)	ECGO	GPE versus GP	308	154	RR, DCR, TTP, SI, AEs
Han BH 2011 [20]	122	35/87	28-78	SCC (37) LAC (78) Others (7)	IIIB (43) IV (79)	ECGO	TCE versus TC + placebo	61	61	RR, DCR, AEs
Zhao X 2011 [21]	69	23/46	35-73	SCC (34) LAC (25) Others(10)	IIIB (11) IV (58)	ECGO	GPE versus GP	33	36	RR, DCR, AEs
Hu HT 2011 [22]	89	21/68	41-70	SCLC (89)	NA	KPS	TPE versus TP	45	44	RR, DCR, AEs
Wen F 2011 [23]	84	27/57	54-75	SCC (36) LAC (44) Others (4)	NA	KPS	NPE versus NP	43	41	RR, DCR, AEs
Zhang H 2011 [24]	56	15/41	36-75	SCC (18) LAC (33) Others (5)	IIIB (32) IV (25)	KPS	NPE versus NP (2) GPE versus GP (15) TPE versus TP (11)	28	28	RR, DCR, TTP, OYS, AEs
Chen Q 2011 [25]	68	27/41	42-75	SCC (20) LAC (44) Others (4)	IIIA (15) IIIB (41) IV (12)	NA	GPE versus GP	33	35	RR, DCR, SI

AEs , adverse effects; DCR, disease control rate; ECGO, eastern cooperative oncology group; F/M, female/male; GP, gemcitabine + cisplatin; GPE, gemcitabine + cisplatin + Endostar; Group 1, Endostar combined with PBDC; Group 2, PBDC alone; KPS, Karnofsky physical status score; LAC, lung adenocarcinoma; N, number of patients; NA, not available; NP, vinorelbine + cisplatin; NPE, vinorelbine + cisplatin + Endostar; OYS, one-year survival rate; PP, paclitaxel + cisplatin; PPE, paclitaxel + cisplatin + Endostar; RR, response rate; RT, radiotherapy; SCC, squamous cell carcinoma; SCLC, small-cell lung cancer; SI, symptom improvement; TC, paclitaxel + carboplatin; TCE, paclitaxel + carboplatin + Endostar; TP, docetaxel + cisplatin; TP, TPE, docetaxel + cisplatin + Endostar; TTP, time to progression;

### Quality of study design

The studies were appraised independently by two authors (Li W and Ming ZJ) based on the criteria from the Cochrane Handbook for Systematic Reviews of Interventions (Version 5.0.1). According to our predefined quality assessment criteria, six of the fifteen trials (40%) were evaluated as having a low risk of bias, and another nine included trials were evaluated as having an unclear risk of bias. Table 2 shows the quality of each study included in the present systematic review.

**Table 2:** Raw data and methodological quality of included trials

**Studies**	**Region**	**Sequence generation**	**Allocation concealment**	**Blind**	**Outcome data**	**Selective outcome reporting**	**Other sources of bias**	**ITT**	**Risk of bias**
Wang JW 2005 [11]	Multi-center	Random number table (SAS)	Insufficient	Clear	No	No	Unclear	Yes	Low risk of bias
Yang L 2005 [12]	Multi-center	Random number table (SPSS)	Unclear	Unclear	Yes	No	Unclear	No	Unclear risk of bias
Cai L 2007 [13]	Single center	Random number table (SPSS)	Unclear	Clear	Yes	No	Unclear	No	Low risk of bias
Mu HY 2009 [14]	Single center	Random number table (SPSS)	Unclear	Clear	Yes	No	Unclear	No	Low risk of bias
Liao HY 2009 [15]	Single center	Random number table (SPSS)	Unclear	Unclear	Yes	No	Unclear	No	Unclear risk of bias
Zhang T 2009 [16]	Single center	Random number table (SPSS)	Unclear	Clear	Yes	No	Unclear	No	Low risk of bias
Liu J 2009 [17]	Single center	Random number table (SPSS)	Unclear	Unclear	Yes	No	Unclear	No	Unclear risk of bias
Ma JB 2009 [18]	Single center	Random number table (SPSS)	Unclear	Unclear	Yes	Yes	Unclear	No	Unclear risk of bias
Shi GY 2009 [19]	Multi-center	Random number table (SAS)	Unclear	Unclear	Yes	No	Unclear	No	Unclear risk of bias
Han BH 2011 [20]	Multi-center	Random number table (SPSS)	Insufficient	Clear	Yes	No	Unclear	No	Low risk of bias
Zhao X 2011 [21]	Single center	Random number table (SPSS)	Unclear	Clear	Yes	No	Unclear	No	Low risk of bias
Hu HT 2011 [22]	Single center	Random number table (SPSS)	Unclear	Unclear	Yes	No	Unclear	No	Unclear risk of bias
Wen F 2011 [23]	Single center	Random number table (SPSS)	Unclear	Unclear	Yes	No	Unclear	No	Unclear risk of bias
Zhang H 2011 [24]	Multi-center	unclear	Unclear	Unclear	Yes	No	Unclear	No	Unclear risk of bias
Chen Q 2011 [25]	Single center	Random number table (SPSS)	Unclear	Unclear	Yes	No	Unclear	No	Unclear risk of bias

ITT, intention-to-treat; SAS, SAS software; SPSS, SPSS software.

### Comparison of ORR between Endostar combined with PBDC and PBDC alone

**Figure 2:**
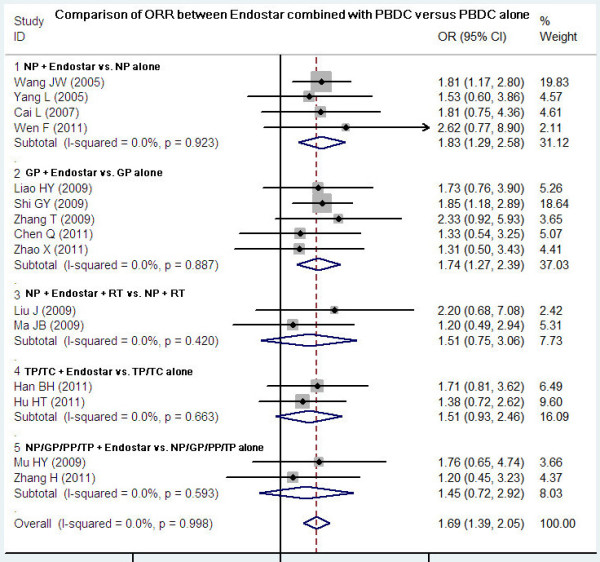
**ORR of Endostar combined with PBDC versus PBDC alone for treating NSCLC.** GP, gemcitabine + cisplatin; NP, vinorelbine + cisplatin; OR, odds ratio; ORR, overall response rate; PBDC, platinum-based doublet chemotherapy; PP, paclitaxel + cisplatin; RT, radiotherapy; TC, paclitaxel + carboplatin; TP, docetaxel + cisplatin.

Fifteen studies compared the ORR between Endostar combined with PBDC and PBDC alone. The results of the fixed effects model showed that OR = 1.69 (95% CI 1.39 to 2.05; test for heterogeneity = 4.53; I^2^ = 0%), test for overall effect: Z = 5.02, *P* < 0.00001. The ORR of Endostar combined with PBDC for treating NSCLC was significantly higher than that of PBDC alone. The subgroup analyses showed that ORR favored the following five Endostar combinations with the overall effect Z-value and *P*-values as follows: NP + Endostar versus NP alone (Z = 4.61, *P* < 0.0001), GP + Endostar versus GP alone (Z = 4.70, *P* < 0.0001), NP + Endostar + radiotherapy versus NP + radiotherapy (Z = 1.93, *P* < 0.05), TP/TC + Endostar versus TP/TC alone (Z = 3.02, *P* < 0.05), and NP/GP/TP/PP + Endostar versus NP/GP/TP/PP alone (Z = 3.48, *P* < 0.05) (Figure 2). Sensitivity analyses showed that the RR and 95% CI did not alter substantially by removing any one trial (data not shown), with an OR pool oscillating between 1.20 and 2.62.

### Comparison of DCR between Endostar combined with PBDC and PBDC alone

**Figure 3:**
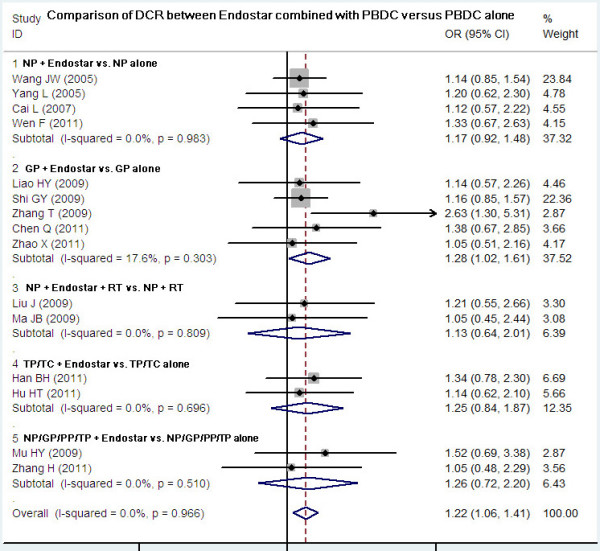
**DCR of Endostar combined with PBDC versus PBDC alone for treating NSCLC.** DCR, disease control rate; GP, gemcitabine + cisplatin; NP, vinorelbine + cisplatin; OR, odds ratio; PBDC, conventional platinum-based doublet chemotherapy; PP, paclitaxel + cisplatin; RT, radiotherapy; TC, paclitaxel + carboplatin; TP, docetaxel + cisplatin.

Fifteen studies compared the DCR between Endostar combined with PBDC and PBDC alone. The results of the fixed effects model showed that the OR was 1.22 (95% CI 1.06 to 1.41; Z = 2.77, *P* = 0.006). The DCR of Endostar combined with PBDC for treating NSCLC was significantly higher than that of PBDC alone. The subgroup analyses showed that DCR favored the following four Endostar combinations with the overall Z-value and *P*-values as follows: NP + Endostar versus NP alone (Z = 3.28, *P* = 0.001), GP + Endostar versus GP alone (Z = 4.64, *P* < 0.0001), TP/TC + Endostar versus TP/TC alone (Z = 3.32, *P* < 0.05), and NP/GP/TP/PP + Endostar versus NP/GP/TP/PP alone (Z = 2.48, *P* < 0.05) (Figure 3). In the analysis of sensitivity, the exclusion of studies individually did not substantially modify the estimators, with an OR pool oscillating between 1.05 and 2.63.

### Comparison of OYS and QOL between Endostar combined with PBDC and PBDC alone

**Figure 4:**
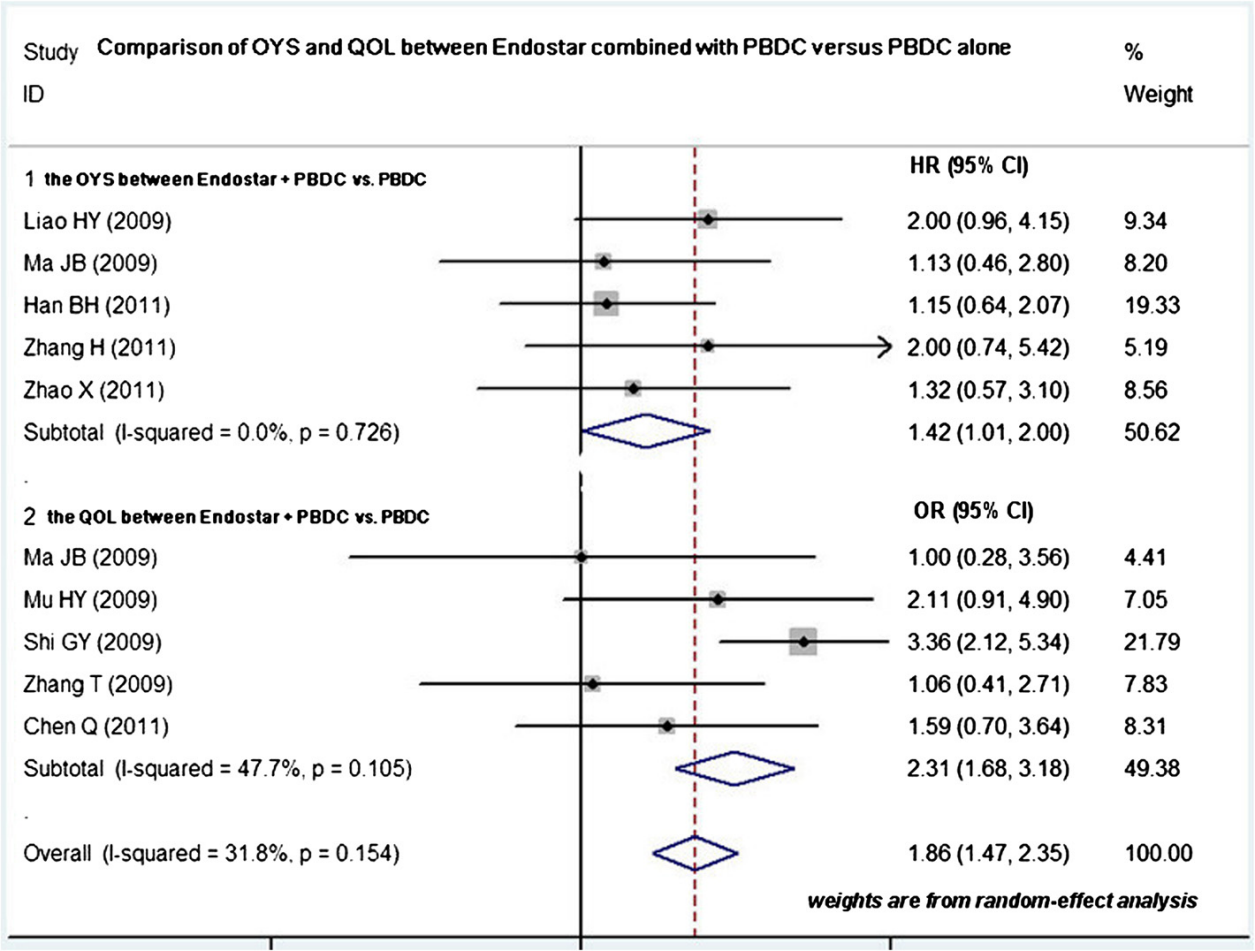
**OYS and QOL improvement of Endostar combined with PBDC versus PBDC alone for treating NSCLC.** HR, hazard ratio; OR, odds ratio; OYS, one-year survival rate; PBDC, conventional platinum-based doublet chemotherapy; QOL, quality of life.

Five studies compared the OYS between Endostar combined with PBDC and PBDC alone [15,18,20,21,24]. The HR was 1.42 (95% CI 1.01 to 2.00; test for heterogeneity = 14.14; I^2^ = 0%), test for overall effect: Z = 5.24, *P* < 0.0001 (Figure 4). The OYS of Endostar combined with PBDC for treating NSCLC was higher than that of PBDC alone. Five trials involving 742 patients compared the QOL between Endostar combined with PBDC and PBDC alone for treating advanced NSCLC [14,16,18,19,25]. The results showed that the OR was 1.86 (95% CI 1.47 to 2.35; test for heterogeneity = 13.19; I^2^ = 47.7%), test for overall effect: Z = 4.65, *P* < 0.00001 (Figure 4). The QOL improvement of Endostar combined with PBDC for treating NSCLC was significantly higher than that of PBDC alone. 

### Comparison of TTP between Endostar combined with PBDC versus PBDC alone

**Figure 5:**
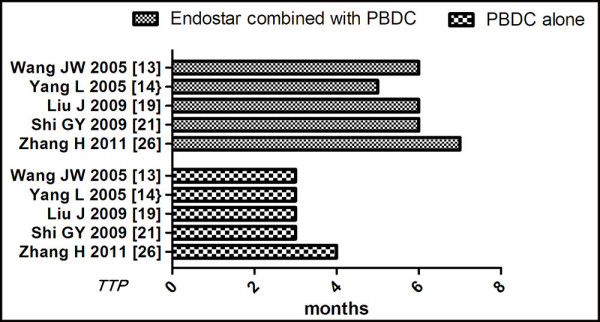
**TTP of Endostar combined with PBDC versus PBDC alone for treating NSCLC.** PBDC, conventional platinum-based doublet chemotherapy; TTP, time to progression.

Five studies reported prolonged TTP for randomized controlled trials of Endostar combined with PBDC versus PBDC alone for treating advanced NSCLC [11,12,17,19,24]. The results showed that the mean ± SD TTP of Endostar combined with PBDC versus PBDC alone was 6.19 ± 0.80 and 3.83 ± 0.73 months, respectively. The t-value was 12.02; the degree of freedom was 4, *P* < 0.00001 (Table 3). The TTP of Endostar combined with PBDC for treating NSCLC was significantly longer than that of PBDC alone (Figure 5). 

**Table 3:** Time to progression of Endostar combined with chemotherapy versus chemotherapy alone for treating NSCLC

	**Endostar combined with PBDC (TTP, months)**	**PBDC alone (TTP, months)**	**T-value**	**95% CI**	***P*-value**
Wang JW 2005 [11]	6.3	3.6	T = 12.02	1.815 to 2.91	0.000
Yang L 2005 [12]	5.03	3.33			
Liu J 2009 [17]	6.2	3.4			
Shi GY 2009 [19]	6.1	3.7	df = 4		
Zhang H 2011 [24]	7.3	5.1			
Mean ± SD	6.19 ± 0.80	3.83 ± 0.73			

95% CI, 95% confidence interval; df, degree of freedom; SD, standard deviation; TTP, time to progression.

### Comparison between Endostar combined with PBDC and PBDC alone by different stratifications

Two trials conducted a statistical analysis of the different stratifications of the patients such as sex, histology, TNM stage, and treatment history. One study [11] indicated that male patients with squamous cell carcinoma and lung adenocarcinoma showed higher RR after receiving Endostar combined with PBDC compared with PBDC alone (*P* = 0.001, *P* = 0.009, *P* = 0.007, respectively). Regardless of whether the patients had a history of treatment, those who received Endostar combined with PBDC had a higher RR (*P* = 0.003, *P* = 0.034, respectively). However, another trial [12] did not find significant differences in those stratifications (Table 4). 

**Table 4:** Comparison of Endostar with PBDC versus PBDC alone by different stratifications

**Item**	**Wang JW 2005**[11]			**Yang L 2005 **[12]		
	**Group 1 N (%)**	**Group 2 N (%)**	***P*-value**	**Group 1 N (%)**	**Group 2 N (%)**	***P*-value**
Sex						
Male	74 (32.3)	19 (16.2)	0.001	-	-	-
Female	40 (43.0)	13 (27.7)	0.08	-	-	-
Histology						
SCC	49 (38.0)	10 (18.2)	0.009	11 (47.8)	3 (30)	0.476
LAC	54 (32.7)	17 (17.3)	0.007	6 (22.2)	5 (21.7)	1.000
Others	11 (39.3)	5 (45.5)	0.72			
TNM stage						
IIIA	17 (33.3)	5 (16.7)	0.11	10 (52.6)	3 (23.1)	0.147
IIIB	29 (33.7)	10 (22.2)	0.17			
IV	68 (36.8)	17 (19.1)	0.003	9 (25.7)	5 (25)	1.000
Treatment history						
No	92 (40.0)	28 (23.9)	0.003	10 (37)	4 (19)	0.214
Yes	22 (23.9)	4 (8.5)	0.034	10 (37)	4 (33.3)	1.000

Group 1, Endostar combined with PBDC; Group 2. PBDC alone; LAC, lung adenocarcinoma; SCC, squamous cell carcinoma.

### Adverse reactions analysis of Endostar combined with PBDC versus PBDC alone

**Figure 6:**
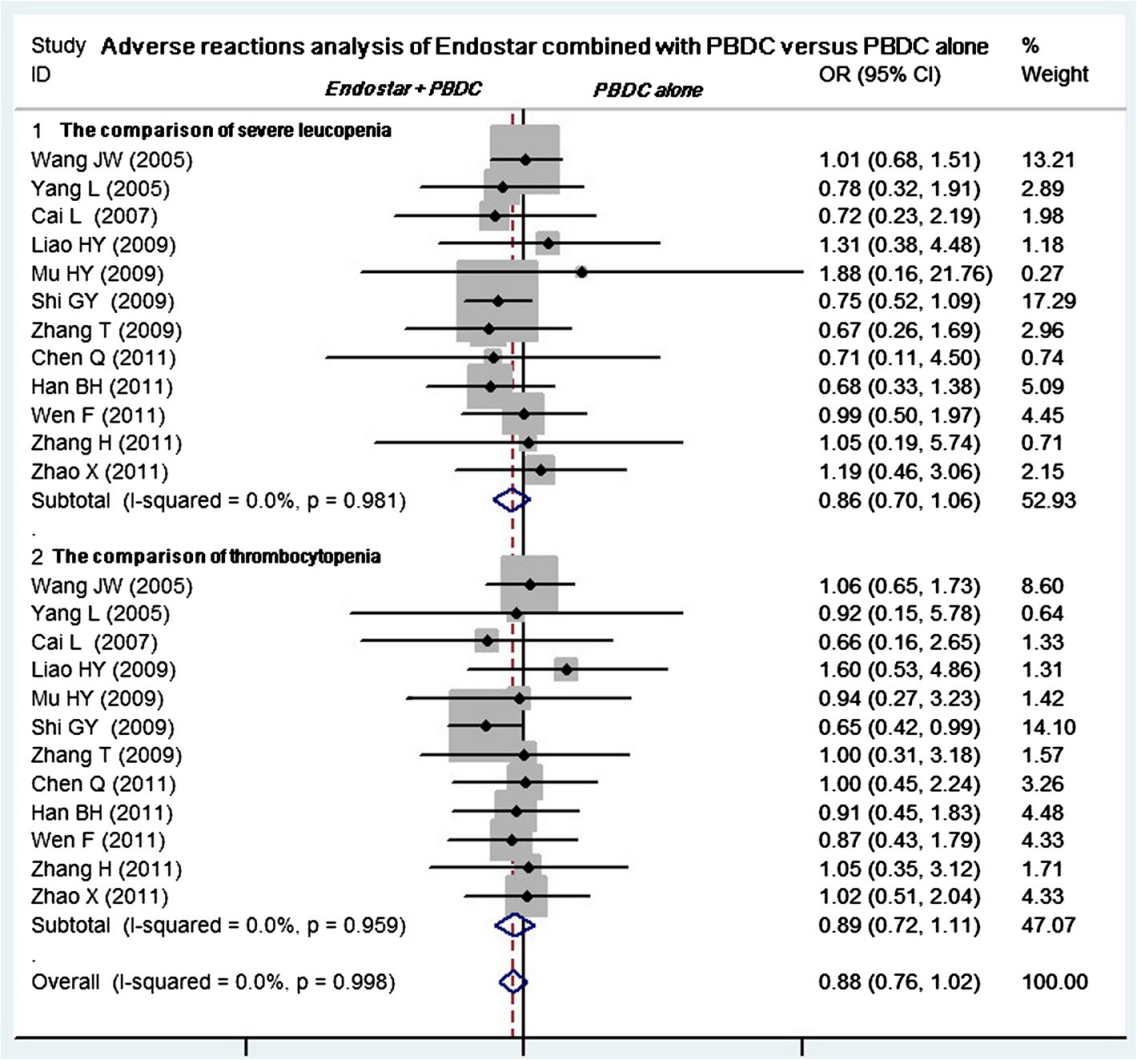
**Meta-analysis of the severe leukopenia and thrombocytopenia between Endostar combined with PBDC and PBDC alone.** OR, odds ratios; PBDC, conventional platinum-based doublet chemotherapy.

**Figure 7:**
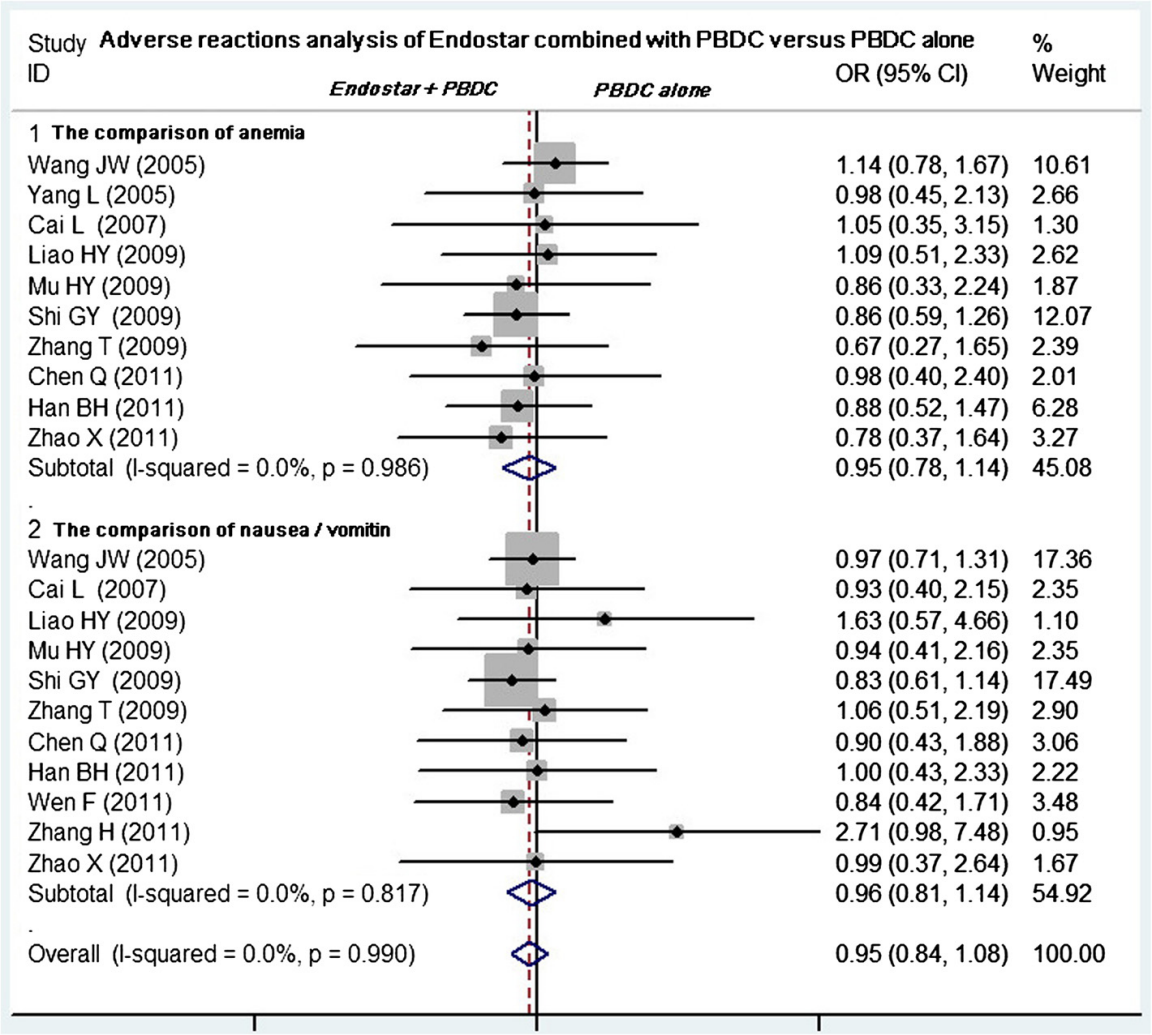
**Meta-analysis of anemia and nausea/vomiting between Endostar combined with PBDC and PBDC alone.** OR, odds ratios; PBDC, conventional platinum-based doublet chemotherapy.

Included trials assessed 11 serious AEs, the most common being gastrointestinal, skin-related, and hematologic diseases. Twelve studies compared the grade 3 or 4 leukopenia and thrombocytopenia between Endostar combined with PBDC and PBDC alone [11-16,19-21,23-25]. The Endostar combination arms had a similar incidence of grade 3 or 4 leukopenia relative to the PBDC arms (OR = 0.86, 95% CI 0.70 to 1.06, *P* = 0.165). No difference in thrombocytopenia incidence was found between Endostar combined with PBDC and PBDC alone (OR = 0.89, 95% CI 0.72 to 1.11, *P* = 0.305) (Figure 6). No significant differences in incidence and severity were found between Endostar combined with PBDC and PBDC alone (Figure 7) in 10 studies comparing anemia [11-16,19-21,25] (OR = 0.95, 95% CI 0.78 to 1.14, *P* = 0.562) and in 11 studies comparing nausea/vomiting [11,13-16,19-21,23-25] (OR = 0.96, 95% CI 0.81 to 1.14, *P* = 0.649). Other common AEs including diarrhea, skin rash, dysfunction of liver, constipation, alopecia, nerve toxicity, and mucositis occurred with similar incidence in the two groups (*P* > 0.05). 

### Analysis of publication bias

**Figure 8:**
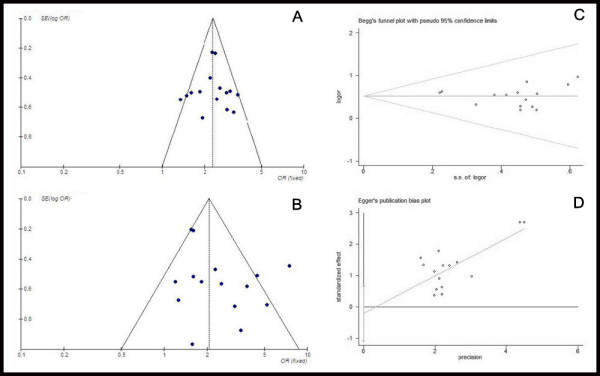
**Assessment of publication bias. (A)** Funnel plot for the ORR of Endostar combined with PBDC versus PBDC alone; **(B)** Funnel plot for the DCR of Endostar combined with PBDC versus PBDC alone; **(C)** Begg’s publication bias plot for the ORR of Endostar combined with PBDC versus PBDC alone; **(D)** Egger’s publication bias plot for the ORR of Endostar combined with PBDC versus PBDC alone.

In the present study, the shape of the funnel plot appeared to be approximately symmetrical and suggested that publication biases may not have a significant effect on the results. The result of the Egger’s test was t = 0.51 (*P* = 0.618), whereas that of the Begg’s test was SD of score = 20.21 (*P* = 0.619). Therefore, both tests suggested that publication biases may not have a significant effect on the results (Figure 8).

## Discussion

The angiogenesis inhibitors for the treatment of cancer as a new approach are based on Folkman’s theory in 1971 [26]. Since then, hundreds of angiogenesis inhibitors were discovered and used in drug development. Endostatin specifically acts on neovascular endothelial cells, inhibits cell migration, and induces cell apoptosis, thus playing a major antiangiogenic role by acting on tumor-associated neovascular endothelial cells [27]. Endostar, a novel recombinant human endostatin expressed and purified in *E. coli* with an additional nine-amino acid sequence and forming another histidine-tag structure, was approved by the SFDA in 2005 for the treatment of NSCLC [8]. In 2005, SFDA licensed Endostar plus NP to treat advanced NSCLC as a first-line therapy. The decision was mainly based on a phase III study [11], which was a randomized, double-blind, multicenter trial comparing treatment with NP plus endostar and NP alone, first-line, in advanced NSCLC patients.

In recent years, several studies have reported on the efficacy and safety of Endostar in the treatment of advanced lung cancer. This systematic review was performed to quantify better the benefits and toxicities of Endostar combined with PBDC versus PBDC alone for treating advanced NSCLC. In this review, 15 reports of randomized trials were identified by searching from the start of each database up to July 2012. A significant benefit of Endostar plus PBDC in ORR was found (OR = 1.69, 95% CI 1.39 to 2.05), translating into a 14.7% (40.3% to 25.6%) absolute improvement. A meta-analysis of DCR was also conducted (OR = 1.22, 95% CI 1.06 to 1.41). A 13.5% improvement (from 64.7% to 78.2%) of Endostar plus PBDC was found compared with PBDC alone. NP plus Endostar versus NP, GP plus Endostar versus GP, NP plus Endostar plus radiotherapy versus NP plus radiotherapy, and TP/TC plus Endostar versus TP/TC showed improvements of 16.5%, 14.7%, 16.7%, and 19.5% in ORR, respectively, and 10.6%, 16%, 9.5%, and 18.2% in DCR, respectively. From the five reports in the present study, the one-year survival rates in the groups of Endostar plus PBDC and the PBDC alone were 55.4% and 45.3%, respectively, reflecting a 10.1% improvement. Five reports analyzed that the TTP of Endostar combined with PBDC (6.19 ± 0.80 months) for treating NSCLC was significantly longer than that of PBDC alone (3.83 ± 0.73 months). However, only five trials providing relative data were included, which were insufficient to reach a decisive conclusion. Therefore, more research is required to gain a clear understanding of the probability. In the E4599 and AVAIL studies, the antiangiogenesis agent bevacizumab plus chemotherapy not only increased ORR, but also improved PFS [28,29]. The combination of rh-endostatin with vinorelbine plus cisplatin or paclitaxel plus carboplatin chemotherapy enhanced the antitumor effect in two large multicenter phase III trials in advanced NSCLC patients [30,31]. The results of present study are consistent with those of reported studies.

The benefit of chemotherapy in incurable cancers needs to be assessed directly through validated health-related QOL instruments, rather than inferred from RRs, survival benefits, and other traditional endpoints [32]. In the present study, 742 eligible patients were enrolled in the assessment of QOL. A significant benefit of Endostar plus PBDC in the overall improvement rate of QOL (OR = 3.93, 95% CI 2.78 to 5.56) was found, translating into a 29.5% (52.3% to 22.8%) absolute improvement. This prospective QOL analysis supports the clinical benefit of the addition of bevacizumab to 5-fluorouracil-based chemotherapy in improving time to disease progression and prolonging overall survival, without compromising the patients’ QOL [32]. In clinical settings, phase I and phase II studies revealed that Endostar was effective as a single agent with good tolerance in pretreated advanced NSCLC patients at a dose of 7.5 mg/m^2^ daily. Special attention should be given to toxic effects typically observed in antiangiogenesis treatment. The AEs found in the present review were mainly hematological reactions, diarrhea, hepatic toxicity, and nausea/vomiting, most of which were grade 1 or 2 and were well tolerated. The results supported that the Endostar combination arms had a similar incidence of AEs compared with PBDC alone. Although no significant differences were found between the two groups, incidences of hematological reactions and nausea/vomiting were slightly higher in the control group (PBDC alone). Whether Endostar combination could relieve the AEs of treatment should be followed up in future studies. Overall, these results indicate that the potential benefit of Endostar may be widely applicable to a patient population closely resembling clinical reality in advanced NSCLC.

Addressing statistical heterogeneity is one of the most important aspects of systematic reviews. The interpretative problems are dependent on heterogeneity because it might affect the conclusions of the meta-analysis. Therefore, heterogeneity among the collection of studies must be quantified. In this review, the included studies were carefully assessed. A good clinical homogeneity was confirmed, and publication bias was not found according to the funnel plot analysis, the Egger’s test, and the Begg’s test. However, some deficiencies in the present meta-analysis were found. First, the quality of subgroup analysis (age, sex, smoking, histology, and treatment status) according to the different agents (Endostar plus PBDC compared with PBDC) was low because the subgroup data were only provided by a few trials. Only two trials fulfilling the subgroup analysis were included, which were insufficient to reach a decisive conclusion. Second, some reports failed to report the method for concealment of allocation, blinding, and ITT. In addition, the partial reports comprise a small sample size, and some of the reports’ experimental control is not very balanced. Most of the included studies were published in Chinese, with heterogeneous data and analysis methods (for example, the different scored scales were used to assess the life quality). In addition, the number of cases available was relatively small. Hence, the validity of the results was compromised. Although such studies were reported to be of low quality, they still contain credible evidence pointing toward such new drugs. Clinical trials are expensive and difficult. Hence, these findings can help choose the most promising agents for study. However, Endostar, as a new strategy, still has many issues to be resolved in further studies. Confirmation of these conclusions in rigorously controlled randomized trials is required before firm conclusions about this therapy can be drawn.

## Conclusion

The results showed that Endostar combined with PBDC was associated with higher ORR, DCR, and TTP as well as superior QOL profiles as compared with PBDC alone. Moreover, Endostar combined with PBDC was shown to slightly decrease AEs. Endostar combined with PBDC exhibited superior efficacy and safety in antiangiogenesis treatment compared with PBDC alone. However, Endostar, as a new strategy, still has many issues to be resolved in further studies. The notable efficacy and activity of Endostar in combination with PBDC suggest that this regimen may have a value in the treatment of previously untreated patients, including those who cannot tolerate more aggressive therapies. However, confirmation of these conclusions in rigorously controlled randomized trials is required before firm conclusions about this therapy can be drawn.

## Competing interests

The authors declare that they have no competing interests.

## Authors’ contributions

Rong BX and Yang SY participated in the design and coordination of the study, carried out the critical appraisal of the studies, statistical analysis of the studies and wrote the manuscript. Li W, Zhang W, and Ming ZJ developed the literature search, carried out the extraction of data, assisted in the critical appraisal of included studies and assisted in writing up. All authors read and approved the final manuscript.
